# A single-center, open-label, randomized, parallel-group trial to pilot the effectiveness of a peer coach behavioral intervention versus an active control in reducing anxiety and depression in patients scheduled for total knee replacement

**DOI:** 10.1186/s12891-023-06460-4

**Published:** 2023-05-05

**Authors:** Assem Jabri, Yuliana Domínguez Páez, Mackenzie Brown, Geyanne Lui, Wai-Kwong Hui, Noelia Hernandez, Michael L. Parks, Alejandro Gonzalez Della Valle, Susan M. Goodman, Samprit Banerjee, Monika M. Safford, Iris Navarro-Millán

**Affiliations:** 1 Division of General Internal Medicine, Weill Cornell Medicine, New York, NY USA; 2 Department of Physical Therapy, Hospital for Special Surgery, New York, NY USA; 3 Department of Orthopedic Surgery, Hospital for Special Surgery, New York, NY USA; 4 Division of Rheumatology, Hospital for Special Surgery, New York, NY USA; 5 Department of Population Health Sciences, Weill Cornell Medicine, New York, NY USA

**Keywords:** Knee osteoarthritis, Knee replacement, Peer coach, Home physical therapy, Cognitive behavioral therapy

## Abstract

**Background:**

*Moving Well* is a behavioral intervention for patients with knee osteoarthritis (KOA) scheduled for a total knee replacement (TKR). The objective of this intervention is to help patients with KOA mentally and physically prepare for and recover from TKR.

**Methods:**

This is an open-label pilot randomized clinical trial that will test the feasibility and effectiveness of the *Moving Well* intervention compared to an attention control group, *Staying Well*, to reduce symptoms of anxiety and depression in patients with KOA undergoing TKR. The *Moving Well* intervention is guided by Social Cognitive Theory. During this 12-week intervention, participants will receive 7 weekly calls before surgery and 5 weekly calls after surgery from a peer coach. During these calls, participants will be coached to use principles of cognitive behavioral therapy (CBT), stress reduction techniques, and will be assigned an online exercise program, and self-monitoring activities to complete on their own time throughout the program. *Staying Well* participants will receive weekly calls of similar duration from research staff to discuss a variety of health topics unrelated to TKR, CBT, or exercise. The primary outcome is the difference in levels of anxiety and/or depression between participants in the *Moving Well* and *Staying Well* groups 6 months after TKR.

**Discussion:**

This study will pilot test the feasibility and effectiveness of *Moving Well*, a peer coach intervention, alongside principles of CBT and home exercise, to help patients with KOA mentally and physically prepare for and recover from TKR.

**Trial registration:**

Clinicaltrials.gov. NCT05217420; Registered: January 31, 2022.

**Supplementary Information:**

The online version contains supplementary material available at 10.1186/s12891-023-06460-4.

## Contributions to the literature

- This clinical trial will pioneer the use of peer coaches for TKR. Peer coaches will have a similar medical profile (age group, history of KOA and TKR) to patients
- This clinical trial will be the first home-based, telephone delivered pre- and post-operative program. The use of the internet and telephone simplifies the logistics of intervention delivery, facilitating wide scalability of the program.
- This clinical trial addresses patient pain catastrophizing, depression, and anxiety through CBT for the first time in a pre- and post-operative program for patients with knee OA undergoing TKR.

## Background

Osteoarthritis (OA) is a common condition affecting an estimated 31 million mostly older US adults between 2008 and 2011 [1]. Knee OA (KOA) can be profoundly debilitating, negatively impacting mobility. Total knee replacement (TKR) is an effective strategy to improve knee function and quality of life, however, up to 30% of patients with TKR continue to experience knee pain after surgery [2–4]. Poor prognostic indicators include obesity, pre-operative deconditioning, high levels of anxiety and/or depression, and pain “catastrophizing” (overemphasis on negative aspects or consequences of an experience) before TKR [5–21]. Yet few interventions have focused on optimizing both mental and physical health in patients with KOA before and after TKR, especially in patients with these risk factors

One approach is to add pre-habilitation (pre-hab) to post-TKR rehabilitation. A meta-analysis of post-TKR interventions did not show differences in self-reported pain > 12 months after TKR, except for one telephone-delivered, home-based intervention of functional exercises delivered by physical therapists, aimed at managing anxiety related to knee pain [22, 23]. This suggests that post-operative interventions alone may be insufficient. However, studies with pre-hab interventions for joint replacement surgery suffer from methodological shortcomings [24, 25]. In a study by Wang et al. on total hip arthroplasty patients [26], the pre-hab intervention group also received an intensive post-operative exercise program, making it difficult to attribute benefits to the pre-hab intervention alone. A consistent challenge in pre-hab programs was low adherence, potentially because they required in-person interaction with the physical therapist [24, 25]. Hence, having a well-designed, feasible intervention, with pre-hab and rehabilitation components that addresses mental and physical health, can serve as a viable alternative to improve post-TKR pain among patients with KOA.

Several studies have shown strong associations between high levels of anxiety, depression, and negative surgery expectations with worse TKR outcomes [5, 16, 20, 21]. These associations were independent of other factors related to persistent pain and low physical function after TKR. Patients with high pain catastrophizing pre-operatively had 2.67 (95% CI, 1.2–6.1) higher odds of < 50% post-operative improvement in pain after adjustment for potential confounders [16]. This evidence emphasizes that in addition to physical health, mental health has a strong influence on TKR outcomes, yet few interventions have targeted these factors.

Cognitive behavioral therapy (CBT) is a type of psychotherapy that teaches patients how to modify dysfunctional thinking and behavior to solve active problems [27, 28]. CBT has been shown to significantly reduce pain, anxiety, depression, and insomnia among patients suffering from headaches, osteoarthritis pain, depression, and insomnia [29–31]. CBT has so far been mainly studied as a way to treat symptoms of OA, and not as a strategy to help patients prepare and recover from TKR [32, 33]. One study that examined pre-TKR CBT for reducing pain catastrophizing and improving pain outcomes after total knee replacement, found no improvement in 3-month pain outcomes after surgery in the intervention group when compared to a non-CBT control [34], indicating that CBT interventions alone may not be sufficient in improving outcomes after TKR.

Peer coaches, also called community health workers, are lay individuals with a specific diagnosis that work with a patient population with the same condition to improve disease management. Peer coaching interventions leverage the interpersonal connection and support between peer and patient to increase patient confidence in achieving favorable outcomes [35]. Studies utilizing peer coaches have demonstrated increased medication adherence among patients with human immunodeficiency virus, asthma, diabetes, and increased cancer screening [36–45]. The social and emotional support peer coaches provide to patients living with the same condition can lead to positive behavior change and engagement and participation in medical decisions related to their condition.

*Moving Well* is a peer-coach delivered intervention that will incorporate pre-habilitation and principles of CBT, with the standard of care for patients undergoing TKR, in order to pilot test the hypothesis that levels of anxiety and depression will be lower in participants in the intervention group, compared to baseline and the attention control group, *Staying Well,* at 6, 12, and 24 months post-TKR. A secondary hypothesis is that participants in the *Moving Well* intervention group will have less knee pain at 6, 12, and 24 months post-TKR compared to the *Staying Well* attention control group.

## Methods

### Theoretical framework and implementation framework

The *Moving Well* peer coach intervention is guided by Social Cognitive Theory (SCT) [35]. This theory posits three mechanisms of human agency: direct personal agency (self-efficacy), proxy agency (reliance on others, such as parents or partners, acting at one’s behest to secure desired outcomes), and collective agency (coordinated interdependent efforts). Table 1 shows how the intervention maps to SCT by listing barriers people with KOA face in the effective participation of their own care and matches these barriers to their related theoretical constructs of SCT. It also maps each *Moving Well* session activity to each construct of the theory. We will use the Reach, Effectiveness, Adoption, Implementation, Maintenance (RE-AIM) framework for the implementation and evaluation of the *Moving Well* intervention [46].

**Table 1:** Moving Well peer coach intervention mapping to social cognitive theory

Theoretical Construct	Targeted Barrier	Intervention Activity	Corresponding Session
**Self-Efficacy**	Anxiety Fear of pain Depressive symptoms Feeling isolated	Positive Thinking Coaching Motivational Interviewing Empowerment	Sessions 1 -7
**Outcome Expectation**	Catastrophizing Unrealistic expectations from total knee replacement	Action planning Motivational Interviewing	Sessions 1—7
**Socio-Cultural Factors**	No place to exercise No partner Lack of understanding of the value of exercise	Supportive coaching Action planning Education (PALS) Exercise program	Sessions 1–12

*PALS* Patient Activated Learning System

### Study population and sample size

The participants of the study will be people with KOA who are 50 years of age or older, have a TKR scheduled in 8 weeks or more, speak English, have access to a computer, the internet, and a working phone. We will exclude people who are unable to exercise (wheelchair-bound or bedbound), have a history of any joint replacement surgery, or have any rheumatic disease other than KOA.

The peer coaches of *Moving Well* will be people with KOA who had a TKR at least 12 months before they initiate their training as peer coaches of the intervention. This study will have 5 peer coaches.

The sample size estimation assumed four repeated measures, a range of intra-class correlations (0.05 to 0.20) for the repeated measures, and a Bonferroni-adjusted overall type I error of 5% (after controlling for two primary outcomes). We estimate a 20% attrition rate and will recruit 93 subjects, resulting in 37 participants in each arm of the study (total of *N* = 74 completers). The trial is designed to have at least 80% power to detect a standardized effect size of d = 0.5 or 0.5 standard deviations between the intervention and attention control arms. The standardized effect size is equivalent to a 2.7-point change in the PHQ-8 score, and a 1.6-point change in the GAD-7 score.

## Trial design

This is an open-label, parallel group, randomized trial that will test the feasibility and effectiveness of a peer coach intervention in lowering the levels of anxiety and/or depression in patients with KOA before and after TKR. All participants will receive the standard of care for patients at the Hospital for Special Surgery (HSS) undergoing TKR, which consists of an educational book and a pre-surgery class. Patients with KOA enrolled in the study will be allocated to either the peer coach intervention (*Moving Well*) group or the attention control (*Staying Well*) group through a block randomization scheme. Participants will receive 50 dollars for every data collection timepoint.

**Fig. 1:**
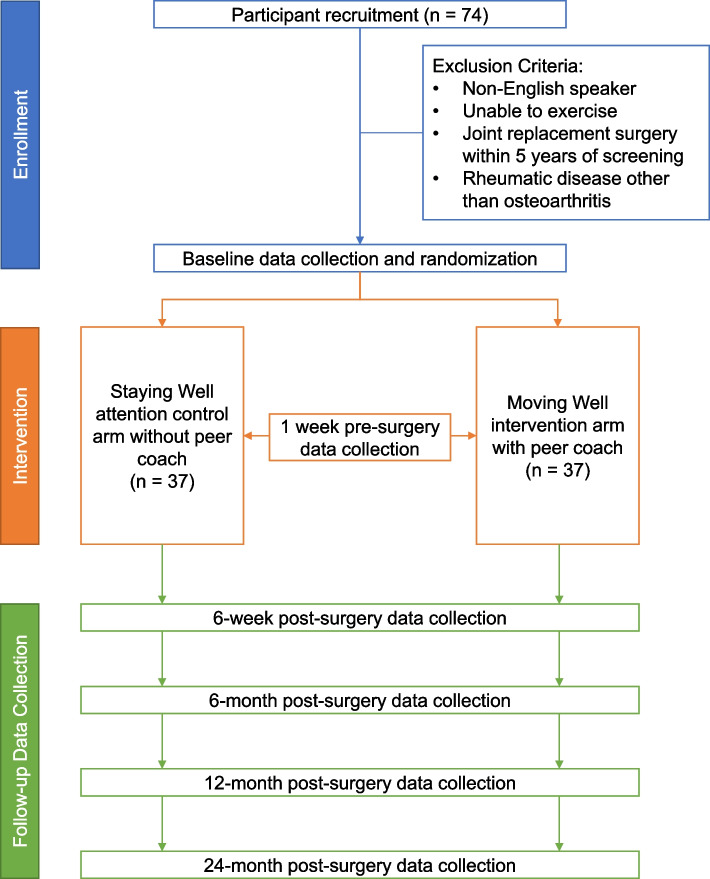
CONSORT diagram

This protocol is written in accordance with guidelines from the CONsolidated Standards of Reporting Trials (CONSORT) (see Fig. 1 for CONSORT) [47], and the Standard Protocol Items: Recommendations for Interventional Trials [48]. The institutional review board from HSS approved this study (protocol number 2019–1298).

### Study interventions

#### Overview of the *Moving Well* intervention

**Fig. 2:**
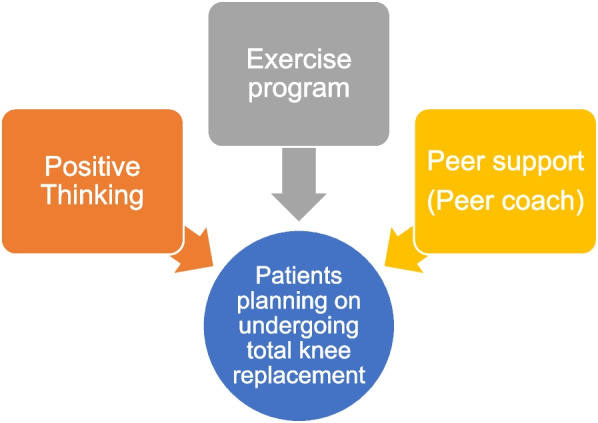
*Moving Well* components

*Moving Well* consists of three main components (Fig. 2): an exercise program delivered through online videos, positive thinking (principle of CBT) training, and peer support with education on TKR, provided through weekly phone sessions between a peer coach and a participant for 12 sessions, 7 sessions in the 7 weeks pre-TKR, and 5 sessions for 5 weeks post-TKR. Table 2 details the curriculum of *Moving Well.* Each session of the intervention is scripted in the peer coach manual with all the content that the peer coach is responsible to deliver during each of the calls. The participant will have an Activity Book, which will contain the same content as the peer coach manual but with tables, and space to complete the daily activities that are assigned to them by the peer coach.

**Table 2:** Moving Well peer coach arm schedule

Session	Objectives	PALS Questions & Answers
1. Introduction to the *Moving Well* Program	1. Introduce the *Moving Well* program 2. Introduce the Patient Activated Learning System (PALS) 3. Discuss the rules and responsibilities of the program 4. Discuss daily exercises	• None
2. Total Knee Replacement, the Relationship Between Mood and Pain, and Daily Exercise	1. Discuss the impact of pain and stress on living a healthy life and the goals of the *Moving Well* program 2. Discuss the **3 Steps** to achieve positive thinking that facilitate living a healthy life 3. Discuss the relationship between mood and pain 4. Discuss daily exercises	• What is osteoarthritis (OA)? • What is a total knee replacement (TKR)? • What is physical therapy? • What are the benefits of exercise? • What is the connection between pain and mood?
3. Negative Thoughts, Stress Reduction, and the Daily Exercise Program	1. Discuss negative thoughts and how to manage them 2. Practice stress reduction techniques 3. Discuss daily exercises	• What can I do about difficult emotions? • What is guided imagery and how can it help me? • How does mindfulness work? • How can I reduce stress? • Review the “Deep Breathing” exercise • Review the “Muscle Relaxation” exercise
4. “Awfulizing”, Physical Activity, Stress, and Pain	1. Introduce “Awfulizing” or “Spiral of Doom” 2. Discuss relationship between physical activity, stress, and pain 3. Discuss daily exercises	• How can I exercise when I’m in pain? • Does exercise help my pain from osteoarthritis (OA)?
5. Social Support and the Daily Exercise Program	1. Discuss social support and the importance of family and friends 2. Discuss daily exercises	• How can family and friends help me recover from total knee replacement (TKR)? • How can I have a better recovery after total knee replacement (TKR)?
6. Keeping Up with the *Moving Well* Steps	1. Discuss monitoring of mood, pain, and exercises 2. Prepare for knee replacement 3. Discuss the study visit 4. Discuss daily exercises	• What can I do about muscle tension?
7. The Safe Use of Opioids and Physical Therapy After Surgery	1. Discuss how to use opioids responsibly 2. Understand the importance of physical therapy after surgery 3. Discuss daily exercises	• How long should I expect to take opioids after my total knee replacement (TKR)? • How important is physical therapy after total knee replacement (TKR)? • How long does it take to recover from total knee replacement (TKR)?
**Surgery**		
8. Post-TKR Recovery and Planning for the Future	1. Discuss plans for knee replacement recovery 2. Discuss *Moving Well* plans for the rest of the program	• None
9. Post-TKR Recovery and Planning for the Future	1. Monitor progress with physical therapy 2. Discuss monitoring of pain, mood, and the 3 steps to achieve positive thinking	• None
10. Post-TKR Recovery and Planning for the Future	1. Monitor progress with physical therapy 2. Discuss monitoring of pain, mood, and the 3 steps to achieve positive thinking	• None
11. Post-TKR Recovery and Planning for the Future	1. Monitor progress with physical therapy 2. Discuss monitoring of pain, mood, and the 3 steps to achieve positive thinking	• None
12. Post-TKR Recovery, Questions, and Closure	1. Monitor progress with physical therapy 2. Discuss monitoring of pain, mood, and the 3 steps to achieve positive thinking 3. Close off the *Moving Well* program and discuss your next steps	• None

#### *Moving Well*: education materials-Patient Activated Learning System (PALS)

The TKR educational materials for the *Moving Well* group are available on the Patient Activated Learning System (PALS) website (www.palsforhealth.com) and are listed in Table 2. The PALS is a publicly available educational and empowerment resource informed by SCT designed to provide engaging, easily understood, and well-researched facts for people who want to know more about health, medicine, and diseases [49]. The content in the PALS is evidence-based and peer-reviewed. This content is translated into patient-facing text in plain language, aiming for a seventh-grade reading level. Some content is accompanied by visuals or short videos and a “sticky soundbite” to reinforce the single learning objective for each module, known in the PALS parlance as a renewable knowledge object (RKO). Each RKO includes an assessment question about the information that the reader has just reviewed.

#### *Moving Well*: exercise program

The pre-and post-surgery exercise program was designed by a certified physical therapist (KWH), who has extensive experience in TKR rehabilitation. The exercise program will be delivered to participants through online videos recorded by certified doctors in physical therapy from Weill Cornell Medicine (WCM). The videos use people with KOA to demonstrate the exercises. The program focuses on strengthening hip, core, and thigh muscles as well as increasing the knee range of motion. The exercise program allows the participant to select an individualized goal (advanced exercises vs. elementary exercises) based on their self-perceived ability.

#### *Moving Well*: principles of cognitive behavioral therapy and positive thinking

During each telephone session, the participants will learn and practice positive thinking, which consists of 3 main steps: *Identify* negative thinking; *Replace* negative thinking with positive thinking; *Practice* positive thoughts and/or healthy behaviors (e.g., exercise, mindfulness) [27, 28]. Each participant is required to monitor their daily mood and negative thoughts, then replace those thoughts with positive thoughts and/or practice positive action. This monitoring process will create self-awareness of their anxiety or depressive symptoms so that they can engage in healthier behaviors. Participants will be instructed on how to perform stress-reduction breathing exercises, meditation, and guided imagery to help manage anxiety and/or depressive symptoms. The CBT component of *Moving Well* will be a facilitator for each participant to decrease their fear of exercising while experiencing pain so that they can increase their self-efficacy in participating in the required, and often challenging, rehabilitation process after TKR.

#### *Moving Well*: peer coach

Peer coaches are not health professionals, they are individuals who have KOA and a history of TKR. Peer coaches will serve as role models and increase participants’ self-efficacy, which is one of the main tenets of the SCT. The role of the peer coach will be to provide support and coaching to each participant so that they successfully complete the activities and assignments of the intervention. The coaching and support by a person who already experienced the same event (had a TKR) will increase the likelihood of behavior change [30, 43, 50, 51]. They will also ensure that participants complete the daily monitoring activities. This will serve to guarantee that participants are completing the activities of the intervention as intended.

The inclusion criteria for peer coaches will be, having KOA and history of TKR at least 12 months before enrollment, and being 60 years of age or older. Once a person who has had TKR meets the criteria to be a peer coach, they will be interviewed by the research team to assess their communication skills. The research team will review each candidate and will document the reasons for not including a candidate as a peer coach. Peer coaches will be considered research subjects and will be consented before starting peer coach training.

#### Peer coach training

Peer coach training is modeled on a previously published approach [52]. Peer coaches will be scheduled for virtual training meetings. There will be a total of 15 web conference training sessions over 6 months, for up to 6 h a week. Peer coaches will be compensated on an hourly basis for the training and all subsequent interactions with the participants in the peer coach intervention group. Table 2 has details of the *Moving Well* curriculum, which is the curriculum that peer coaches will be trained on during the 6-month virtual training.

Before each training session, peer coaches-in-training will need to review the learning materials on PALS, listen to recordings of mock sessions between a peer coach and a participant, review the *Moving Well* participant Activity Book, and review the relevant session in the Peer Coach Manual. During training meetings, peer coaches will receive brief didactic education on the content of the *Moving Well*session assigned for the day, listen to mock sessions, practice delivering the session with a partner, and discuss their performance with the research team and other peer coaches [53]. Every week, peer coaches in training will be paired to practice delivering the session covered in the latest conference on their own time, in preparation for certification of that session. During the certification session, a coach-in-training will deliver the assigned session to a member of the research team over the phone, who will play the role of the participant. The research team will use a checklist to assess proficiency and communication skills during the call (Supplementary File 1). Peer coaches will be approved as peer coaches by members of the research team once they receive scores of 90% or more in all sessions, and each coach will have an opportunity to re-take the certification session if they do not achieve the required score. All peer coaches must pass all certification sessions to be able to work with a participant of *Moving Well.*

Peer coach training will also include two Motivational Interviewing (MoI) skills training meetings with the research team and only two coaches at a time. Each MoI training meeting will include the following activities: 1) Practicing MoI skills using role-playing scripts where coaches will alternate their roles between coach and participant under the supervision of the research team. 2) Reinforcing skills with live feedback and encouragement, allowing peer coaches to critique each other’s skills and propose ways for improvement on techniques like “rolling with resistance”, “action planning”, and the use of the MoI-style open-ended questions, affirmations, reflective listening, and summaries (OARS).

#### Peer coach retention

The strategies that we will use to retain coaches include ensuring timely payment for work and continuing education/training with opportunities for practicing skills. Ongoing support will be provided through weekly group conference calls outside of training time. These conference calls are used to problem-solve any challenges encountered, make sure they receive the support that they need to excel as peer coaches, and help build group identity within the peer coaches.

#### *Staying Well*: attention control arm

The subjects in the *Staying Well* attention control group will receive 12 weekly calls (7 before and 5 after TKR) from research assistants. These calls will be similar in length to those of *Moving Well* and cover topics not related to those of *Moving Well or TKR* (Table 3). These attention calls will help determine if providing a patient with attention and information not directly related to TKR and rehabilitation will yield the same outcomes as providing specific information and guidance for TKR.

**Table 3:** Staying Well attention control arm schedule

Session	Objectives	Health Topics
**Session 1**	A. Overview of the goals and objectives of the program B. Get to know each other	Introduction to the Program
**Session 2**	A. To learn how to read nutritional facts labels B. To discuss the importance of fruits and vegetables	Healthy Eating and You: Part 1 a. Reading Nutritional Facts Label^a^ b. Portion Sizes^a^ c. Fruits and Vegetables^a^ d. Making Healthy Food Choices^a^ e. What is a healthy diet?
**Session 3**	A. Learn about heart-healthy fats and proteins B. Learn about grains and dairy C. Learn about fiber rich food	Healthy Eating and You: Part 2 a. Healthy Fats vs. Less Healthy Fats^a^ b. Powered by Protein^a^ c. Meat, Heart disease, and Cancer^a^ d. Plant-Based Protein^a^ e. Grains and Dairy^a^ Fiber-rich Foods
**Session 4**	A. To learn discuss how to put into practice what you have learned about healthy eating B. To learn about healthy eating outside of the home C. To discuss recipe modifications for heart healthy recipes	Healthy Eating and You: Part 3 a. Practice Healthy Eating b. Healthy Eating Outside of the Home^a^ c. Social Events and Your Health^a^ d. Healthy Recipe Modifications^a^
**Session 5**	A. To learn about added sugars B. To learn about sugar-sweetened beverages To learn about menu planning	Healthy Eating and You: Part 4 a. Drinking Water^a^ b. Sugar-sweetened Beverages^a^ c. Sports drinks and energy drinks^a^ d. Added Sugar^a^ e. Snacks and Treats^a^ Menu Planning^a^
**Session 6**	A. To learn the symptoms of high blood pressure B. To provide an overview of the treatment for high blood pressure	High Blood Pressure a. What is high blood pressure? b. Symptoms of high blood pressure c. Treatments for high blood pressure d. Salt and high blood pressure Blood pressure and my body
**Session 7**	A. To learn about good and bad cholesterol B. To learn how and when to treat high cholesterol	Cholesterol a. What is cholesterol? b. Good vs. Bad Cholesterol c. Health Complications with High Cholesterol d. Treatments for High Cholesterol e. Atherosclerotic Cardiovascular Disease (ASCVD) risk, Heart Failure, and Stroke f. Getting a cholesterol test
**Session 9**	A. To learn about the different doctors that care for you (primary care doctor and specialist) B. To learn about disease prevention	Your Doctors and You a. What is a primary care provider? b. What is a specialist or specialty doctor? The importance of yearly checkups
**Session 10**	A. To learn about cancer prevention and early detection	Cancer Screenings a. Colorectal Cancer b. Cervical Cancer c. Prostate Cancer Breast Cancer
**Session 11**	A. To learn about the harmful effects of tobacco use B. To learn about secondhand smoking and it’s affects C. To learn about the cancers caused by smoking	Tobacco Use a. Tobacco Use Screening b. Tobacco Alternatives c. Secondhand smoking d. Lung cancer screening Smoking related cancers
**Session 12**	A. To review the health risk of alcohol consumption	Alcohol and My Health a. Alcohol consumption recommendations b. Alcohol Misuse c. Health Risks of Drinking Alcohol Interactions Between Medicines and Alcohol

^a^Content adapted from Rebecca A. Seguin-Fowler, PhD, RDN, CSCS on behalf of the StrongPeople Program (formerly known as the StrongWomen Program)

#### Guidelines for surgery cancellation, postponement, and concomitant care

Participants in both arms of the study will be free to withdraw at any time. In the event of total knee replacement surgery postponement, the participant will be allowed a maximum 2-week interruption in the study schedule. If there is a more than 2-week delay in surgery and therefore a greater than 2-week gap in the study schedule, or the surgery is cancelled, the reason/s for postponement or cancellation will be collected, and the participant will undergo an exit interview, and no further data will be collected. Participants who have taken part in an exit interview will be allowed to rejoin the study later if they wish to, and continue in their previous intervention allocation, however, they must start from the beginning of the intervention schedule. Participants in either arm of the study are permitted to receive any concomitant care related to their knee OA or TKR while in the study.

### Participant recruitment, enrollment, and randomization

Participants will be recruited from the orthopedic clinics at HSS in Manhattan, New York. The target sample will be 74 patients. To enroll 74 participants, we expect to screen 500 individuals with KOA who are scheduled to undergo TKR. We will monitor recruitment success including screen failure rates, to inform the design of the planned larger study to follow this one. Patients who meet the inclusion criteria will be contacted by research staff and invited to join the study. Once a patient meets the criteria and we have obtained informed consent, they will be scheduled for their first data collection visit, either in person or virtually, with a blinded research assistant, and subsequently randomized. Block randomization will be used in REDCap to randomize participants to either the *Moving Well* arm or the *Staying Well* arm.

### Primary and secondary outcomes

The primary outcome will be the difference in levels of anxiety and depression 6-, 12-, and 24-months post-surgery between *Moving Well* and *Staying Well*. The GAD-7 [54] and PHQ-8 [55] will be used to assess the participants’ level of anxiety and depression respectively. Participants will complete the data collection survey at baseline, 6-, 12-, and 24-months post-surgery. If participants have not completed the data collection surveys, a blinded member of the research team will follow up with a phone call to complete the forms over the phone and minimize missing data.

Secondary outcomes will include change from baseline to 6-, 12-, and 24-months post-surgery in the level of social support, general health status, level of pain catastrophizing, knee pain and function, level of self-efficacy, level of sleep disturbance, and opioid use for knee pain. Table 4 has details of the instruments that we will be using to measure primary and secondary outcomes and the time points that data collection will occur. These secondary outcomes will be measured respectively using the following: Lubben Social Network Scale-18 (LSNS-18) [56], 12-Item Short Form Survey (SF-12) [57], Pain Catastrophizing Scale (PCS) [58], Knee Injury and Osteoarthritis Outcome Score (KOOS) Pain and Activities of Daily Living (ADL) sub-scales [59], General Self-Efficacy Scale (GSF) [60], Patient-Reported Outcomes Information System (PROMIS) Sleep Disturbance Scale [61], and participant self-report use of opioids for knee pain.

**Table 4:** Data collection timepoints and respective outcomes

	**Baseline**	**1-week pre- surgery**	**6-weeks post- surgery**	**6-months post-surgery**	**1-year post- surgery**	**2-years post-surgery**
Sociodemographic information (collected via EHR and patient survey)	x					
Medical history (collected via EHR and patient survey)	x					
Surgical history (collected via EHR and patient survey)	x					
**Primary Outcomes – Patient-reported**						
PHQ-8 (depression)	x	x	x	x	x	x
GAD-7 (anxiety)	x	x	x	x	x	x
**Secondary Outcomes – Patient-reported and/or electronic health record**						
Resources used to prepare for surgery	x	x				
KOOS pain and ADL subscales	x	x	x	x	x	x
Lubben Social Network Scale	x	x	x	x	x	x
Short Form-12	x	x	x	x	x	x
Pain Catastrophizing Scale	x	x	x	x	x	x
General Self-Efficacy Scale	x	x	x	x	x	x
Opioid use	x	x	x	x	x	x
COVID-19 screener survey	x	x	x	x		
Exercise survey	x	x	x	x	x	x
Sleep disturbance (PROMIS)	x	x	x	x	x	x
Surgical outcomes			x	x	x	x
Program Evaluation			x	x	x	x
Knee range of motion	x	x	x	x		
Blood Pressure	x	x	x	x		
Resting Heart Rate	x	x	x	x		
Weight	x	x	x	x		
Timed up and go test	x	x	x	x		
6-min walk test	x	x	x	x		
30 second chair stand test	x	x	x	x		
Quadriceps Strength using a handheld dynamometer	x	x	x	x		

*EHR* Electronic health record, *PHQ – 8* Patient Health Questionnaire 8 items, *GAD-7* Generalized Anxiety Disorder 7 items, *KOOS ADL* Knee Injury and Osteoarthritis Outcome Score Activity of Daily Living Sub-scale, *COVID-19* Coronavirus disease 2019, *PROMIS* Patient-Reported Outcomes Measurement Information System

Other secondary outcomes include the post-surgery inpatient rehabilitation duration assessed by participant self-report at 6 months post-surgery, and change in the following objectively measured physical parameters from baseline to 6 months post-surgery: knee range of motion using a goniometer, Timed Up and Go test (TUG), 6 Minute Walk Test (6MWT), 30-Second Chair Stand test, and quadriceps strength using a handheld dynamometer (only for those that did the data collection in person) [62–64]. Table 5 contains information about the measures that will be collected in the study with the corresponding time points.

**Table 5:** Detailed measures table

Name/Construct	Type (i.e., primary, secondary, other)	Time Frame	Description / Instrument
**Patient Health Questionnaire-8 (PHQ-8)** [55]	Primary	Baseline and 6-month follow up	The PHQ-8 is an 8-item scale that screens for depression. Scores range from 0 to 24 with higher scores indicating greater severity of depression
**Generalized Anxiety Disorder Assessment (GAD-7)** [54]	Primary	Baseline and 6-month follow up	The GAD-7 is a 7 item self-reported questionnaire for screening and severity measuring of generalized anxiety disorder. Scores range from 0 to 21, with higher scores indicating greater severity of anxiety
**Knee Injury and Osteoarthritis Outcome Score (KOOS) pain and function in daily living sub-scales** [59]	Secondary	Baseline and 6-month follow up	The KOOS pain sub-scale is a 9-item scale that assesses knee pain. Scores range from 0 to 100 with lower scores indicating greater severity of pain. The KOOS function sub-scale is a 17-item scale that assesses function. Scores range from 0 to 100 with lower scores indicating worse knee function
**Lubben Social Network Scale-18 (LSNS-18)** [56]	Secondary	Baseline and 6-month follow up	The LSNS-18 is an 18-item scale that measures perceived social support. Scores range from 0 to 90 with higher scores indicating greater social engagement
**12-Item Short Form Survey (SF-12)** [57]	Secondary	Baseline and 6-month follow up	The SF-12 is a 12-item scale that assesses general health status. Scores range from 0 to 100 with higher scores indicating better physical and mental health functioning
**Pain Catastrophizing Scale (PCS)** [58]	Secondary	Baseline and 6-month follow up	The PCS is a 13-item scale that assesses the level of catastrophic thinking related to an individual's pain. Scores range from 0 to 52 with higher scores indicating a higher level of pain-related anxiety
**General Self-Efficacy (GSE)** [60]	Secondary	Baseline and 6-month follow up	General self-efficacy (GSE) is a reliable and valid instrument to assess this disposition. This scale is a self-report measure of self-efficacy of 10 items. The total score ranges between 10 and 40, with a higher score indicating more self-efficacy
**Opioid use**	Secondary	Baseline and 6-month follow up	Self-reported use of opioids for knee pain
**Patient-Reported Outcomes Information System (PROMIS) Sleep Disturbance Scale** [61]	Secondary	Baseline and 6-month follow up	The PROMIS Sleep Disturbance Scale is a 8 item scale that measure self-reported perceptions of sleep quality, depth, and restoration within the past seven days. Scores range from with higher scores indicating greater sleep disturbances
**Surgical outcome**	Secondary	6-month follow up	Self-reported duration of inpatient rehabilitation
**Physical measurements**	Secondary	Baseline and 6-month follow up	Physical measurements will be collected by a blinded researcher in-person and from the electronic health record: Knee range of motion using a goniometer, blood pressure, resting heart rate, weight, time up and go test, 6-min walk test, 30 s chair to stand test, and quadriceps strength using a handheld dynamometer
**Program Evaluation**	Other	6-month follow up or after dropping out from the study	All participants will complete a program evaluation survey at the end of their time with the study or at the time they drop out
**Demographics**	Other	Baseline	Age, sex, race/ethnicity, education, insurance, income, work status, marital status
**Medical/Surgical history**	Other	Baseline	Diagnosed conditions, medications/treatments, previous joint replacement surgeries
**Social history**	Other	Baseline	Smoking, vaping, alcohol consumption history
**Implementation climate** [46]	Feasibility	6-month follow-up	We will determine the number of participants that completed the study in the attention control arm and Moving Well intervention arm and compare with how many initiate the study. The intervention will be considered feasible if more than 80% of enrolled participants complete the intervention and data collection points Retention of peer coaches in the study of 80% or more will be another metric that we will use for feasibility
**Implementation and Evaluation climate** [46]	Reach	Baseline	We will collect demographic and social data on all the participants that join the intervention. We will store the demographic and social information of individuals that drop out from the study. This information will provide us with an understanding of how the intervention is reaching a diverse group of people with rheumatoid arthritis
**Implementation and Evaluation climate** [46]	Adoption	At 6-month follow-up data collection or after dropping out from the study	We will collect demographic and social data among all participants. We will also be inviting both participants that completed the study and those that drop out, to participate in semi-structured interviews regarding their experience with the study, the Moving Well curriculum, and working with a peer coach. This information will allow us to improve the adoption of the intervention to a larger clinical trial
**Implementation and Evaluation climate** [46]	Implementation/ Fidelity	Calls of sessions 1–12 between peer coaches/research staff and participants	All calls between peer coaches and participants will be recorded and reviewed by the research team to assess fidelity of the intervention Peer coaches must have discussed at least 80% of the items in the checklist of each of the sessions to make sure that the intervention is been delivered as intended. We will do additional training for peer coaches who are completing less than 80% of the corresponding session checklist
**Implementation and Evaluation climate** [46]	Maintenance	Weekly throughout the study (weekly calls with peer coaches) and after 6-month follow up data collection	We will conduct semi-structured interviews to determine the best way to support participants and peer coaches, to maximize retention for the Moving Well program, and receive feedback for the intervention

### Implementation and program evaluation

We will use the RE-AIM implementation and program evaluation framework for this study. We will track the number of participants that complete the study in the peer coach intervention arm and control arm. The feasibility of the study will be assessed by examining the completion of the program (80% of enrolled participants complete the intervention), and satisfaction among participants and peer coaches. We will perform one-sample tests on program adherence and high satisfaction in the *Moving Well* arm by comparing these outcomes with an 80% benchmark.

Demographics of eligible individuals who chose not to participate will be compared to those who did choose to participate to estimate the magnitude of selection bias and to gain an understanding of how the intervention is reaching a diverse group of people with KOA scheduled for TKR. Other feasibility outcomes include the completion rate of the intervention, duration of the phone calls between coaches and clients, sustainability of peer coaches’ network, and retention of peer coaches.

We will invite participants that completed the study and those that drop out to participate in semi-structured interviews regarding their experience with the study. This information will allow us to adapt the intervention for use in in a larger clinical trial. All calls between peer coaches and clients will be recorded and reviewed by the research team to assess the fidelity of the intervention. Peer coaches must have discussed at least 80% of the items in the checklist of each of the sessions to make sure that the intervention is been delivered as intended. We will do additional training for peer coaches that are completing less than 80% of the corresponding session checklist. Table 5 details the implementation and evaluation procedures of the intervention.

### Data analysis

We will use descriptive statistics, t-tests, and Chi-square tests as appropriate to compare patients in each study arm. We will assess group differences between baseline and the week before TKR, 6 weeks after TKR, and 6-, 12-, and 24-months after TKR with parametric and nonparametric analyses including one-sample t-tests, Wilcoxon rank sum, and Chi-square tests or Fisher’s exact test of proportions, as appropriate. All tests will be two-sided (unless otherwise stated) and the overall type I error will be maintained at 5%.

We will use linear and generalized linear mixed effects regression models to analyze the repeated measures of all outcomes. Although a significant treatment*time interaction will conclude rejection of the hypothesis, in this pilot study we will focus on obtaining estimates of baseline adjusted treatment effects at the end of treatment as preliminary evidence of effectiveness to power a future trial. We will correct the inflation of the type I error due to multiple primary outcomes using Holm’s stepdown procedure. Due to the exploratory nature of the secondary outcomes, we will not adjust the type I error. Our mixed models provide valid inferences when data are missing at random. However, we will also test the plausibility of this assumption and repeat our analysis using pattern-mixture models [65].

### Data management

All data will be collected using REDCap. Research staff will code all data fields to identify queries generated by REDCap and address them appropriately. Research staff will receive a unique login and password for the REDCap system and will be able to view and verify data accuracy. All participant data will be stored on secure HSS and WCM network servers.

### Monitoring

As this study is a behavioral intervention with a small sample size, a data monitoring committee will not be used. Interim analyses will not be conducted.

### Ethics and dissemination

Important protocol modifications will be communicated to all relevant parties including investigators, trial participants, regulatory bodies, and the trial registry. The research team will protect participant confidentiality by: (i) removing direct identifiers from stored information [(i.e., names, social security numbers, medical record numbers)]; (ii) securing and limiting access to information that would identify participants; and (iii) limiting access to information stored to HSS and WCM investigators. Data will be stored on password protected network servers at HSS and WCM and the use of REDCap ensures the secure collection of data from participants. Peer coaches will not be collecting any data and will receive appropriate protected health information training.

The final data set will be stripped of identifiers prior to release for sharing. Study data and associated documentation will be made available to researchers after approval from respective institutional review boards and under the following rules: a) the requestor shall provide resources for data transfer; b) the data is used for research purposes only; c) appropriate data security measures are taken by the requester. Trial results will be available to participants, healthcare professionals, and the public through press release and publications.

## Discussion

The *Moving Well* intervention is a highly innovative intervention that aims to improve mental and physical health before and after TKR. In this pilot, we will be studying the effect of *Moving Well* on mediating factors of pain and low self-efficacy, high levels of anxiety, and depression. Once we have tested the feasibility of *Moving Well* and pilot-tested the effectiveness of the intervention, we will then prepare for a large-scale multi-site clinical trial to test its effectiveness in decreasing post-TKR pain in a fully powered trial.

*Moving Well* was adapted from the *Living Healthy* intervention [50], which was a peer coach intervention for patients with diabetes to improve musculoskeletal pain. As demonstrated by the original *Living Healthy *intervention trial, peer coaches can enhance adherence with home exercises and physical activity [50]. Peer coaches can serve to overcome the shortcomings of pre-hab programs. These shortcomings include the requirement for in-person physical therapy, which limits adherence to the pre-hab program, and increased cost of the intervention due to having an actual physical therapist to conduct the exercises [24, 25, 66]. The lower cost of peer coaches could help with the scale-up of physical therapist-delivered interventions, which can be too costly. Similarly, private insurance companies, including Medicaid, are covering costs for peer coach services, which will serve to support the scale-up of the intervention and enable it to be available to everyone beyond research purposes [67, 68].

Several studies have shown strong associations between anxiety, depression, and procedure expectations with TKR outcomes [5, 15, 19–21]. These associations were independent of other factors associated with persistent pain and low physical function after TKR. As previous studies have shown that physical pre-habilitation alone is not enough to improve post-TKR pain, *Moving Well*will target anxiety, depressive symptoms, and physical fitness both before and after TKR. The pre-TKR program focuses on increasing participants’ self-efficacy in performing rehabilitation exercises with the goal of decreasing their fear of exercise because of knee pain after their TKR. This mental preparation will consist of participants using principles of CBT, increasing their self-awareness on how their pain affects their mood and vice versa. This will be followed by creating strategies to change these disruptive thoughts or behaviors so that they can increase their self-efficacy in engaging in physical activity and rehabilitation exercises despite their pain. At the same time, a systematic review showed that the professional qualifications of trained coaches delivering structured online programs based on these principles are of minor importance in terms of the efficacy of these interventions [69]. Hence, the rationale for the use of peer coaches in the *Moving Well* intervention.

### Innovations

The innovations of this study include 1) the heavy emphasis on patient-centeredness and a multi-stakeholder participatory model for the design and development of all materials; 2) the use of peer coaches for patients scheduled to undergo TKR; 3) the use of principles of CBT (positive thinking) to prepare for and recover from TKR; 4) A pre-surgery at-home exercise program; 5) employment of cost-effective personnel (peer coaches), overcoming a significant barrier for the scale-up of physical therapist-delivered interventions.

The intervention will be designed to be patient friendly as it is being developed with the direct input of the peer coaches who have a history of knee OA and TKR. These innovative aspects of this project could change the current treatment paradigm for patients undergoing TKR.

### Strengths and limitations

The strengths of this study include the evidence-based nature of the *Moving Well* peer coach intervention. Another strength is that it is an adaptation of a previously effective intervention called *Living Healthy,* whose investigators are part of this team (MMS). *Moving Well* is informed by a theoretical framework (Social Cognitive Theory) as described previously, and the use of an implementation framework (RE-AIM) to guide evaluation will increases the likelihood of effectiveness and implementation. Participants recruited for the study will include those of low socioeconomic status defined as being beneficiaries of Medicaid or on no insurance, and those from underrepresented racial/ethnic groups, which will potentially reveal barriers to the scale-up of this intervention and guide future clinical trial design.

Limitations include the small sample size, although this is to be expected in a pilot study. Another limitation is the requirement for participants to speak English, and have access to a computer, the internet, and a working phone, which means that non-English speaking patients from minority populations, or those who can’t afford a computer, phone, or access to the internet will not be recruited. This may impact the generalizability of the results of the intervention. However, future scale-up of the clinical trial will include translated intervention materials in Spanish and loan internet-equipped devices for participants without internet to be able to access all online materials.

## Conclusion

This highly innovative study will help develop a foundation for a future larger trial that can leverage significant strategies to improve the preparation and recovery from TKR surgery. It can also help in the development of future interventions targeted at patients managing their chronic KOA pain who are not candidates for TKR or that are not interested in the procedure. Finally, we expect that it will be of general help in addressing health care challenges for people with chronic diseases such as KOA.

## Supplementary Information

**Additional file 1: **Peer Coach Evaluation Checklist.

## Data Availability

Data for the study design can be found in the manuscript figures, tables, and supplementary materials. For further information on study design, please email the corresponding author, Iris Navarro-Millán, MD MSPH at yin9003@med.cornell.edu.

## Abbreviations

6MWT: 6 Minute Walk Test
ADL: Activities of Daily Living
CBT: Cognitive Behavioral Therapy
CONSORT: CONsolidated Standards of Reporting Trials
GAD-7: Generalized Anxiety Disorder-7
GSE: General Self-Efficacy Scale
HSS: Hospital for Special Surgery
KOA: Knee Osteoarthritis
KOOS: Knee Osteoarthritis Outcome Score
KOOS-ADL: Knee Osteoarthritis Outcome Score-Activities of Daily Living
LSNS-18: Lubben Social Network Scale-18
MoI: Motivational Interviewing
OA: Osteoarthritis
OARS: Open-ended questions, Affirmation, Reflective Listening, Summary
PALS: Patient Activated Learning System
PCS: Pain Catastrophizing Scale
PHQ-8: Patient Health Questionnaire-8
Pre-hab: Prehabilitation
PROMIS: Patient-Reported Outcomes Information System
RE-AIM: Reach, Effectiveness, Adoption, Implementation, Maintenance
REDCap: Research Electronic Data Capture
RKO: Renewable Knowledge Objects
SCT: Social Cognitive Theory
SF-12: 12-Item Short Form Survey
TKR: Total Knee Replacement
TUG: Timed Up and Go test
WCM: Weill Cornell Medicine
