# Supplementary material for: A single-center, open-label, randomized, parallel-group trial to pilot the effectiveness of a peer coach behavioral intervention versus an active control in reducing anxiety and depression in patients scheduled for total knee replacement
Source: BMC Musculoskelet Disord. 2023 May 5;24:353. doi: 10.1186/s12891-023-06460-4 (PMC10160708; doi:10.1186/s12891-023-06460-4)
Supplement: Supplementary file 1 — Additional file 1: Peer Coach Evaluation Checklist. [file 12891_2023_6460_MOESM1_ESM.pdf]

PEER COACH: **DATE:** **SESSION#:** **ATTEMPTS #:**

PARTICIPANT: **EVALUATORS:**

SESSION CONTENT (40%)

**Did the peer coach display the following competencies?**	**No**	**Needs Improvement**	**Yes**
Greetings between peer coach and participant	○	○	○
Introduction to session	○	○	○
Summarize previous session	○	○	○
Review session assignments	○	○	○
Review monitoring table for negative thoughts, pain, and mood	○	○	○
Review monitoring table for deep breathing exercises	○	○	○
Review monitoring table for physical exercises	○	○	○
Discuss session content	○	○	○
Discuss assignments for next session	○	○	○
Schedule next session call	○	○	○
	○	○	○

OARS EVALUATION (20%)

**Did the peer coach display the following competencies?**	**No**	**Needs Improvement**	**Yes**
**OPEN-ENDED QUESTIONS**	○	○	○
Did the coach ask open-ended questions?	○	○	○
Did the coach keep communication moving forward?	○	○	○

**AFFIRMATIONS**	◯	◯	◯
Did the coach provide affirmations towards the participant?	◯	◯	◯
Were the coach’s affirmations sincere?	◯	◯	◯
Did the coach acknowledge and validate the participant’s experience and feelings?	◯	◯	◯
**REFLECTIVE LISTENING**	◯	◯	◯
Did the coach demonstrate reflective listening?	◯	◯	◯
Did the coach ask questions instead of assuming they understood the participant?	◯	◯	◯
Did the coach show they had interest and respect for what the participant had to say?	◯	◯	◯
Did the coach demonstrate that they accurately heard and understood the participant?	◯	◯	◯
**SUMMARIZING**	◯	◯	◯
Did the coach summarize and reinforce main points of the conversation	◯	◯	◯
Did the coach show they have been listening to the participant?	◯	◯	◯
Did the coach smoothly transition to the next topic?	◯	◯	◯

GENERAL MANNER (40%)

	**No**	**Needs Improvement**	**Yes**
Did the coach arrive to their certification session on time?	◯	◯	◯
Was the coach supportive to their participant?	◯	◯	◯
Was the coach kind to their participant?	◯	◯	◯
Did the coach connect well with their participant?	◯	◯	◯

What were the coach's strengths during this certification session?

What are some areas the coach can improve upon from this certification session?

What feedback does the coach have for the study team? This includes feedback on training, study materials, or the program in general.

Did the coach pass this certification session? ☐
